# Thirty-day readmissions due to Venous thromboembolism in patients discharged with syncope

**DOI:** 10.1371/journal.pone.0230859

**Published:** 2020-04-13

**Authors:** Sudeep K. Siddappa Malleshappa, Gautam K. Valecha, Tapan Mehta, Smit Patel, Smith Giri, Roy E. Smith, Rahul A. Parikh, Kathan Mehta

**Affiliations:** 1 Division of Hematology-Oncology, Baystate Medical Center, Springfield, MA, United States of America; 2 Department of Medicine, Staten Island University Hospital, Staten Island, NY, United States of America; 3 Department of Neurology, University of Minnesota, Minneapolis, MN, United States of America; 4 Division of Neurology, University of Connecticut, Hartford, CT, United States of America; 5 Division of Hematology-Oncology, Yale New Haven Hospital, New Haven, CT, United States of America; 6 Division of Hematology-Oncology, University of Pittsburgh Medical Center, Pittsburgh, PA, United States of America; 7 Division of Hematology-Oncology, University of Kansas Medical Center, Kansas City, KS, United States of America; Maastricht University Medical Center, NETHERLANDS

## Abstract

A recent study found that approximately 1 in every 6 patients hospitalized for the 1st episode of syncope had an underlying pulmonary embolism (PE). As current guidelines do not strongly emphasize evaluation for PE in the workup of syncope, we hypothesize that there might be a higher rate of 30-day readmission due to untreated venous thromboembolism (VTE). The objective of this study is to measure the 30-day readmission rate due to VTE and identify predictors of 30-day readmission with VTE among syncope patients. We identified patients admitted with syncope with ICD9 diagnoses code 780.2 in the Nationwide Readmission Database (NRD-2013), Healthcare Cost and Utilization Project (HCUP). The 30-day readmission rate was calculated using methods described by HCUP. Logistic-regression was used to identify predictors of 30-day readmission with VTE. Discharge weights provided by HCUP were used to generate national estimates. In 2013, NRD included 207,339 eligible patients admitted with syncope. The prevalence rates of PE and DVT were 1.1% and 1.4%, respectively. At least one syncope associated condition was present in 60.9% of the patients. Among the patients who were not diagnosed with VTE during index admission for syncope (N = 188,015), 30-day readmission rate with VTE was 0.5% (0.2% with PE and 0.4% with DVT). In conclusion, low prevalence of VTE in patients with syncope and extremely low 30-day readmission rate with VTE argues against missed diagnoses of VTE in index admission for syncope. These results warrant further studies to determine clinical impact of work up for PE in syncope patients without risk factors.

## Introduction

Syncope refers to transient loss of consciousness that is characterized by rapid onset, short duration (few seconds), and spontaneous resolution. It is related to temporary cerebral hypoperfusion. Syncope is an atypical presentation of acute pulmonary embolism (PE). It is usually seen in association with central and/or massive PE [1–3]. The occlusion of >50% of pulmonary vascular bed can result in acute right ventricular failure followed by impaired left ventricular filling, diminished cardiac output, hypotension, and cerebral hypoperfusion [1]. However, the occurrence of syncope in the setting of non-massive PE may be explained by other pathophysiology such as hemodynamically unstable dysrhythmia and vasovagal reflex [4]. Recently published report found that approximately one of every six patients (17.3%) hospitalized for the first episode of syncope had underlying PE [5]. As current international guidelines do not strongly emphasize on evaluation for PE in the work up of syncope [6–8], it is possible that several syncope patients with undiagnosed PE could have been discharged from the hospital without any treatment for their PE. Hence, we hypothesize that syncope patients discharged without diagnoses of venous thromboembolism (VTE) could have high 30-day readmission rates due to untreated VTE. The objective of this study is to measure 30-day readmission rate due to VTE and identify predictors of 30-day readmission with VTE among syncope patients.

## Materials and methods

We identified patients admitted with syncope with ICD9 diagnoses code 780.2 in the Nationwide Readmission Database (NRD-2013), Healthcare Cost and Utilization Project (HCUP), Agency for Healthcare Research and Quality [9]. Patients with PE were identified by ICD9 diagnoses code 415.1[10]. Patients below18 years of age, pregnant patients, patients on anticoagulants and patients with elective 30 day readmission were excluded. Other syncope associated conditions were identified by ICD-9 codes. All the ICD-9 Codes used in analysis are provided in S1 Table. Deyo’s modification of Charlson’s Co-morbidity Index (CCI) was used to quantify severity of co-morbid conditions [11]. The primary outcome was 30 day unplanned readmission rate with VTE, including PE and deep vein thrombosis (DVT) in syncope patients discharged without diagnoses of VTE during index hospitalization. The 30 day readmission rate was calculated using methods described by HCUP[12]. Logistic regression was used to identify predictors of 30 day readmission with VTE. Discharge weights provided by HCUP were used to generate national estimates. Using SAS 9.3, SURVEY procedures with WEIGHT, STRATA, and CLUSTER statements were used to adjust for stratified cluster design of NRD. We accessed and analyzed these data in compliance with the Health Insurance Portability and Accountability Act of 1996, so this protocol is exempted from the institutional review board’s approval at University of Pittsburgh Medical Center. A flow chart on study design is provided in S1 Fig.

## Results

In 2013, NRD included 207,339 eligible patients admitted with syncope. The prevalence rates of PE and DVT were 1.1% and 1.4%, respectively. At least one syncope associated condition was present in 60.9% of the patients. 39.1% of patients had no apparent cause of syncope upon discharge.

Among the patients who were not diagnosed with VTE during index admission for syncope (N = 188,015), 30 day readmission rate with VTE was 0.5% (0.2% with PE and 0.4% with DVT). Among the patients diagnosed with or without syncope associated conditions on index admission, 30 day readmission rate with VTE was 0.6% and 0.4%, respectively. The differences in the baseline characteristics of syncope patients with and without 30 day readmission with VTE are shown in Table 1. A higher proportion of patients with 30 day readmission with VTE were males (52.2% vs. 46.8%, p = 0.004). Patients with 30 day readmission with VTE had higher burden of co-morbid conditions (CCI 2.34 vs. 1.51, p<0.0001) compared to patients without 30 day readmission with VTE; including higher age, obesity, heart and/or respiratory failure, rheumatologic disorder and/or acute infection, active cancer, decreased mobility and recent trauma or surgery (Table 1). The patients with 30 day readmission with VTE had a higher length of stay in index admission (5.58 days vs 3.82 days, p<0.0001) and higher rates of discharge with home health care (22.1% vs. 14.33%, p<0.0001) or discharge to facility (27.3% vs. 16.9%, p<0.0001) compared to patients without 30 day readmission with VTE.

**Table 1:** Baseline characteristics of 30-day readmissions in syncope patients with Venous Thromboembolism (VTE) (N = 188,015).

Characteristics	Syncope with VTE patients with 30 day readmission (N = 919)	Syncope with VTE patients without 30 day readmission (N = 187,096)	P-value
Gender (%)			0.0044
Male	52.15	46.75	
Female	47.85	53.26	
Charlson/Deyo comorbidity index (mean ± SE)	2.34±0.09	1.51± 0.01	< .0001
Comorbidities (%)			
Age ≥ 70 years	56.88	52.66	0.0417
Obesity	11.99	9.38	0.0139
Hormonal Therapy	0.08	0.06	0.84
Heart and/or respiratory failure	23.85	16.29	< .0001
Ischemic stroke or acute myocardial infarction	13.43	11.12	0.055
Acute rheumatologic disorder and/or acute infection	40.67	33.11	< .0001
Active cancer	15.75	5.00	< .0001
Previous VTE	10.46	2.57	< .0001
Decreased mobility	3.07	1.98	0.0399
History of Thrombophilia	0.55	2.00	0.066
Previous trauma /surgery within last month	28.95	20.76	< .0001
Median household income category for patient's zip code (percentile)			0.93
0-25^th^	28.72	29.48	
26-50^th^	26.06	26.16	
51-75^th^	24.34	23.22	
76-100^th^	20.88	21.14	
Primary Payer (%)			0.0004
Medicare	70.97	64.53	
Medicaid	8.65	8.39	
Private including HMO	14.75	18.89	
Self-pay/no charge/other	5.64	8.19	
Hospital characteristics (%)			
Hospital bed size			0.0069
Small	9.48	12.10	
Medium	22.14	25.03	
Large	68.38	62.87	
Hospital location/teaching status (%)			0.0005
Metropolitan non-teaching	40.62	40.77	
Metropolitan teaching	52.62	48.06	
Non-metropolitan hospital	6.76	11.17	
Admission type (%)			0.20
Non-Elective	93.05	94.13	
Elective	6.95	5.87	
Admission day (%)			0.74
Weekday	75.04	74.49	
Weekend	24.96	25.51	
Length of stay (Means ± SE)	5.58±0.20	3.82±0.03	< .0001
Disposition (%)			< .0001
Home	49.49	66.75	
Home health care	22.09	14.33	
Facility	27.34	16.94	
Against medical advice	1.08	1.99	

^#^ Values are numbers (percentages) unless otherwise indicated.

Multivariable predictors of 30-day readmissions with VTE in syncope patients are listed in Table 2. Factors associated with higher 30-day readmissions with VTE included history of previous VTE (OR: 3.74; p<0.0001), active cancer (OR: 3.08; p<0.0001), obesity (OR: 1.29; p = 0.025), trauma or surgery in last 1 month (OR: 1.32; p = 0.002), respiratory and/or heart failure (OR: 1.29; p = 0.004), acute rheumatologic disorder and/or acute infection (OR: 1.18; p = 0.036), large bed size hospital (OR: 1.35, p = 0.006), discharge to facility (OR: 1.94, p<0.0001), and home health care (OR: 1.78, p = 0.001). Factors associated with lower 30-day readmissions with VTE included female sex (OR: 0.78, p = 0.002) and non-metropolitan hospital (OR: 0.62, p = 0.002). Traditional risk factors such as age above 70 years, history of thrombophilia, or hormonal therapy use did not appear to increase the risk of 30-day readmission for VTE.

**Table 2:** Multivariate predictors of 30-days unplanned readmission with Venous thromboembolism in syncope patients (N = 188,015, weighted N = 419,276).

Predictors	Odds Ratio (95% Confidence Interval)	P-value^#^
Age ≥ 70 years	0.89 (0.72–1.10)	0.27
Male	Ref	
Female	0.78 (0.67–0.91)	0.002
Hospital type		
Metropolitan non-teaching hospital	Ref	
Metropolitan teaching hospital	1.03 (0.89–1.20)	0.69
Non-metropolitan hospital	0.62 (0.46–0.85)	0.002
Hospital bed size		
Small	Ref	
Medium	1.13 (0.84–1.51)	0.78
Large^®^	1.35 (1.04–1.75)	0.006
Discharge type		
Routine	Ref	
Discharge to short term hospital, SNF*, ICF*	1.94 (1.59–2.38)	< .0001
Home health care	1.78 (1.44–2.19)	0.001
Against medical advice	0.69 (0.37–1.28)	0.23
Hospital zip code income quartile (In percentile)		
0-25^th^	Ref	
26-50^th^	1.00 (0.81–1.24)	0.93
51-75^th^	1.03 (0.80–1.32)	0.66
76-100^th^^β^	0.95 (0.76–1.18)	0.47
Admission type		
Elective	1.07 (0.80–1.43)	0.64
Weekend	0.99 (0.83–1.19)	0.95
Comorbidities		
Obesity	1.29 (1.03–1.61)	0.025
Heart and/or respiratory failure	1.29 (1.08–1.53)	0.004
Acute rheumatologic disorder and/or acute infection	1.18 (1.01–1.37)	0.036
Previous trauma or surgery within that last month	1.32 (1.10–1.57)	0.002
Active cancer	3.08 (2.48–3.84)	< .0001
Previous history of VTE	3.74 (2.94–4.76)	< .0001
Hormonal therapy	1.62 (0.22–12.03)	0.64
Ischemic stroke or acute myocardial infarction	1.10 (0.88–1.37)	0.41
Decreased mobility	1.18 (0.76–1.86)	0.46
History of thrombophilia	1.49 (0.48–4.61)	0.49

*SNF = Skilled nursing facility; ICF = Intermediate care facility

β Median household income for patient's ZIP Code

® Bedsize of hospital-derived from https://www.hcup-us.ahrq.gov/db/vars/hosp_bedsize/nrdnote.jsp

^#^ P value at 95% confidence interval.

## Discussion

Our study has demonstrated that the prevalence of PE is low in patients presenting with syncope using a large nationwide database compared to the recently published report [5] and similar to other studies[13, 14]. Our study also showed that a majority of patients had at least one syncope associated diagnosis at index admission, and one should consider PE as an etiology if syncope is unexplained[15]. More recently, Costantino et al. have shown a similar low prevalence rate of PE of less than 3% in hospitalized patients and less than 1% within 90 days of follow-up [16]. Moreover, the 30 day readmission rate with VTE was also significantly low in patients with and without syncope associated diagnoses during the index admission. Another study by Epstein et al. have shown that at 3 year follow up in anticoagulant naïve patients, the cumulative incidence of VTE was low at 1.9%, indicating that PE is an uncommon cause of syncope [17]. Since NRD does not capture death outside the hospital, it is possible that a certain number of patients may have died after discharge due to untreated PE. However, extremely low readmission rates due to VTE argue against missed diagnoses of clinically significant PE during their index admission. PE is an uncommon cause of syncope and would need a high index of clinical suspicion before considering appropriate work up[6, 18]. These results warrant a prospective investigation regarding relevant workup for PE in patients admitted for syncope, which might include using pretest probability score like PERC Rule or Wells Criteria, appropriate use of D-dimer testing and considering further imaging based on the results [19–21]. It might be more reasonable to consider VTE work up in syncope patients with hemodynamic instability [15, 22].

The syncope patients with other risk factors for VTE (history of previous VTE, active cancer, obesity, trauma or surgery in last 1 month, respiratory and/or heart failure, and acute rheumatologic disorder and/or acute infection) are at higher risk for 30 day readmission with VTE. This may indicate that among these patients, diagnoses of VTE may have been missed during the index admission for syncope. Hence, a higher index of suspicion for VTE is necessary for patients presenting with syncope that has these risk factors.

Our study has several limitations. The NRD does not contain patient identifiers, and it is not possible to confirm the listed diagnoses. Since the NRD does not contain data on vital signs, laboratory values, radiological data, EKG, or specific medications, we are unable to assess the severity of VTE. We are unable to capture patients with VTE who are treated as an outpatient after discharge. However, given the large sample size, we believe that the estimates of the prevalence of VTE in syncope patients, 30-day readmission rate with VTE, and predictors of 30 day readmission with VTE are reliable.

In conclusion, the prevalence of diagnosed VTE is low in patients with syncope. An extremely low 30 day readmission rate with VTE argues against missed diagnoses of VTE. A higher index of suspicion for VTE is necessary for patients presenting with syncope that has specific risk factors for VTE.

## Supporting information

S1 Fig Study design. (TIFF)

S1 Table ICD-9 codes used in analysis. (DOCX)
