# Supplementary figures and images for: Thirty-day readmissions due to Venous thromboembolism in patients discharged with syncope
Source: PLoS One. 2020 Apr 13;15(4):e0230859. doi: 10.1371/journal.pone.0230859 (PMC7153877; doi:10.1371/journal.pone.0230859)

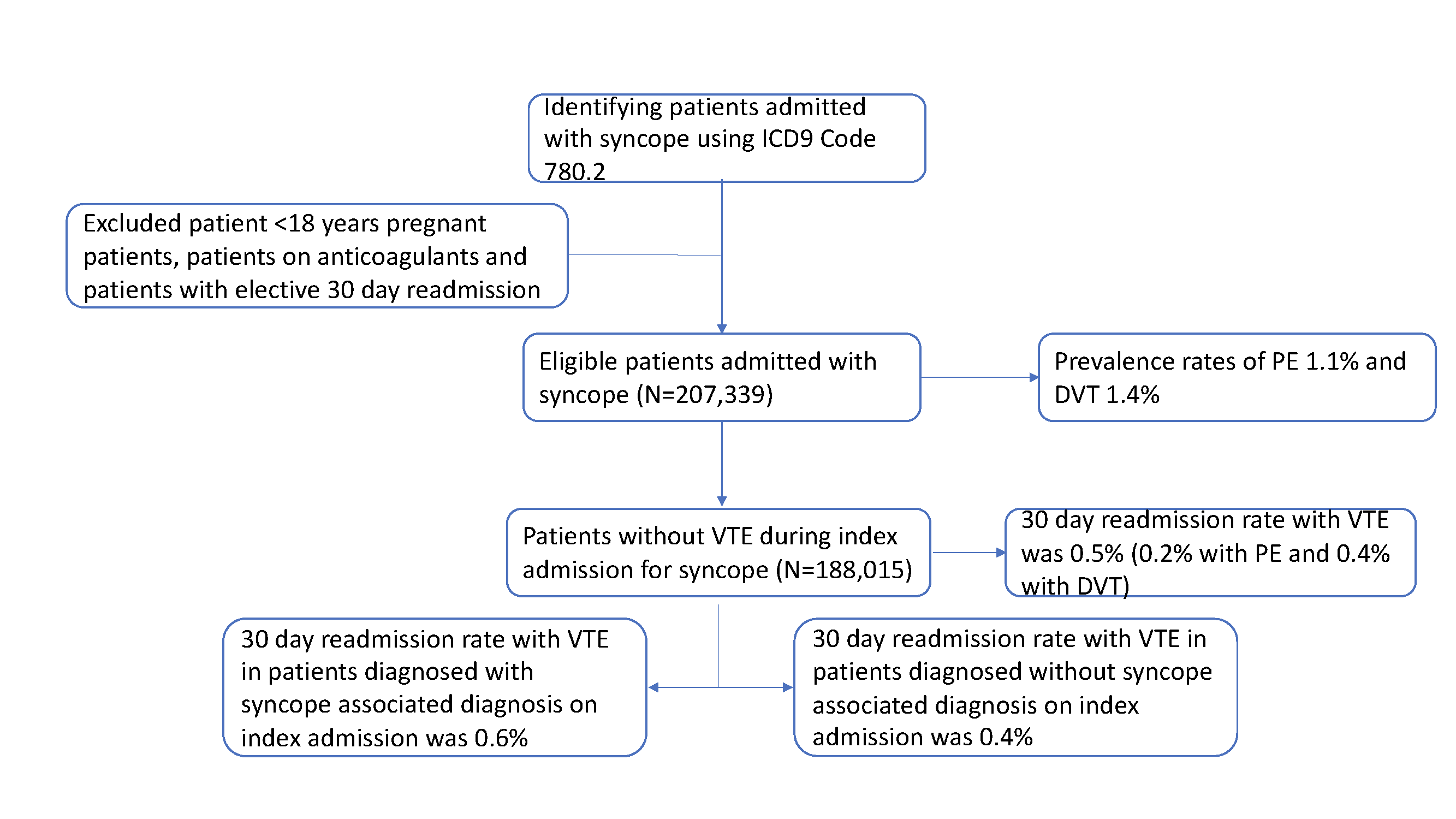

Supplement: S1 Fig — (TIFF) [file pone.0230859.s001.tiff]
