# Supplementary material for: Thirty-day readmissions due to Venous thromboembolism in patients discharged with syncope
Source: PLoS One. 2020 Apr 13;15(4):e0230859. doi: 10.1371/journal.pone.0230859 (PMC7153877; doi:10.1371/journal.pone.0230859)
Supplement: S1 Table — (DOCX) [file pone.0230859.s002.docx]

| S1 Table: ICD-9 codes used in analysis | |
| --- | --- |
| **Causes of syncope** | |
| **Cardiac causes of syncope** | |
| Arrhythmia | 426, 427, 996.01, 996.04 |
| Hypertrophic cardiomyopathy | 425.1 |
| Structural disease(cardiac) | 395, 396, 410, 424.1, 424.3 |
| Structural disease (other) | 415.1, 441.0, 416.0, 423.3 |
| **Neurally mediated (reflex) syncope** | |
| Carotid sinus syndrome | 337.01 |
| **Orthostatic hypotension-mediated syncope** | |
| Hypotension | 458 |
| Volume depletion | 276.5 |
| Autonomic dysreflexia | 337.3 |
| Gastrointestinal (GI) bleeding | Upper or lower GI bleed as  below + 578.1, 578.9 |
| Upper gastrointestinal bleeding | 456.0, 530.7, 578.0, 456.20,  530.21, 530.82, 531.00, 531.01,  531.20, 531.21, 532.00, 532.01,  532.20, 532.21 , 533.00, 533.01,  533.20, 533.21, 534.00, 534.01,  534.20, 534.21, 531.40, 531.41,  531.60, 531.61, 533.40, 533.41,  533.60, 533.61, 534.40, 534.41,  534.60, 534.61, 535.01, 535.11,  535.41, 535.51, 535.61, 537.83 |
| Variceal bleeding | 456.0, 456.20 |
| Lower gastrointestinal bleeding | 569.3, 562.12, 562.13, 562.02,  562.03, 569.85, 569.86 |
| Hematochezia | 569.3 |
| **Neurologic causes of syncope** | |
| Ischemic Stroke | 433.0, 433.3 |
| Intracranial haemorrhage | 430, 431, 432 |
| Transient ischemic attack | 435 |
| Epilepsy | 345 |
| **Other causes of syncope** | |
| Heat syncope | 992.1 |
| Hypoglycemia | 250.8 |
| **ICD9 codes for other variables used in analysis** | |
| Deep vein thrombosis | 451, 452, 453 |
| Long term current use of anticoagulants | V58.61 |
| Pregnancy | Diagnoses codes: 630 – 669, V27  Procedure codes: 72 – 75 |
